# Collaborative goal setting with elderly patients with chronic disease or multimorbidity: a systematic review

**DOI:** 10.1186/s12877-017-0534-0

**Published:** 2017-07-31

**Authors:** Neeltje P. C. A. Vermunt, Mirjam Harmsen, Gert P. Westert, Marcel G. M. Olde Rikkert, Marjan J. Faber

**Affiliations:** 1 Radboud university medical center, Radboud Institute for Health Sciences, Scientific Center for Quality of Healthcare (IQ healthcare), PO Box 9101, NL-6500 HB Nijmegen, Netherlands; 2 The Dutch Council for Health and Society (Raad voor Volksgezondheid en Samenleving, RVS), PO Box 19404, NL-2500 CK The Hague, Netherlands; 3 Department of Geriatrics, Radboud university medical center/Radboud Alzheimer Center, PO Box 9101, NL-6500 HB Nijmegen, Netherlands

**Keywords:** Health priority setting, Goal setting, Decision-making, Chronic health condition, Systematic review, Multifactorial intervention, Multimorbidity, Elderly

## Abstract

**Background:**

It is challenging to use shared decision-making with patients who have a chronic health condition or, especially, multimorbidity. A patient-goal-oriented approach can thus be beneficial. This study aims to identify and evaluate studies on the effects of interventions that support collaborative goal setting or health priority setting compared to usual care for elderly people with a chronic health condition or multimorbidity.

**Methods:**

This systematic review was based on EPOC, PRISMA and MOOSE guidelines. Pubmed, PsychInfo, CINAHL, Web of Science, Embase and the Cochrane Central Register of Controlled Trials were searched systematically. The following eligibility criteria were applied: 1. Randomised (cluster) controlled trials, non-randomised controlled trials, controlled before-after studies, interrupted time series or repeated measures study design; 2. Single intervention directed specifically at collaborative goal setting or health priority setting or a multifactorial intervention including these elements; 3. Study population of patients with multimorbidity or at least one chronic disease (mean age ± standard deviation (SD) incl. age 65). 4. Studies reporting on outcome measures reducible to outcomes for collaborative goal setting or health priority setting.

**Results:**

A narrative analysis was performed. Eight articles describing five unique interventions, including four cluster randomised controlled trials and one randomised controlled trial, were identified. Four intervention studies, representing 904, 183, 387 and 1921 patients respectively, were multifactorial and showed statistically significant effects on the application of goal setting (Patient Assessment of Chronic Illness Care (PACIC) goal setting subscale), the number of advance directives or the inclusion of goals in care plans. Explicit attention for goal setting or priority setting by a professional was a common element in these multifactorial interventions. One study, which implemented a single-factor intervention on 322 patients, did not have significant effects on doctor-patient agreement. All the studies had methodological concerns in varying degrees.

**Conclusions:**

Collaborative goal setting and/or priority setting can probably best be integrated in complex care interventions. Further research should determine the mix of essential elements in a multifactorial intervention to provide recommendations for daily practice. In addition, the necessity of methodological innovation and the application of mixed evaluation models must be highlighted to deal with the complexity of goal setting and/or priority setting intervention studies.

**Electronic supplementary material:**

The online version of this article (doi:10.1186/s12877-017-0534-0) contains supplementary material, which is available to authorized users.

## Background

The number of morbidities and especially the proportion of patients suffering from multimorbidity increase with age. A cross-sectional study of one-third of the Scottish population found that half of the population suffered from at least one morbidity by the age of 50 and most people were multimorbid by the age of 65 [1]. Chronic health conditions and multimorbidity (i.e. the coexistence of two or more chronic morbidities) are challenges in the decision-making process between practitioners and patients. A patient-goal-oriented approach to health care could be beneficial and contribute to a patient’s wellbeing and quality of life [1–4].

However, goal setting and/or priority setting with elderly patients within the framework of a chronic health condition or multimorbidity is complex. Disease-specific guidelines are often not applicable to elderly patients with multiple conditions [5]. Health-related goals can arise from a variety of dimensions [6, 7]. Moreover, care-related goals for community-dwelling frail older adults differ between individuals and often also cover wellbeing, just as much as health and functioning [8]. These different types of goals, which are often implicit, can conflict [9]. In addition, a patient and a physician can also have competing priorities [5, 10]. Therefore, practitioners need approaches for revealing and reconciling their own and their patients’ priorities. However, the availability and effects of approaches for reconciling clinicians’ own and their patients’ priorities and setting goals are not yet clear [5]. Collaborative goal setting, defined as ‘a process by which health care professionals and patients agree on a health-related goal’ [11], could be useful for personalising care and adapting it to a patient’s goals, values and resources. Systematic reviews have been conducted on (collaborative) goal setting in varying rehabilitation settings [12–15]. To our knowledge, however, there has not yet been a systematic review of the effects of interventions supporting collaborative goal setting and/or priority setting for the population of older patients with a chronic health condition or multimorbidity independent of setting. Therefore, we aim to systematically review the availability and effects of interventions supporting collaborative goal setting and/or priority setting compared to the usual care for elderly people with a chronic health condition or multimorbidity.

## Methods

This review was developed and conducted based on the Effective Practice and Organization of Care Cochrane collaboration guidelines (EPOC), Preferred Reporting Items for Systematic Reviews and Meta-Analyses (PRISMA) and Meta-analysis Of Observational Studies in Epidemiology (MOOSE) guidelines ([16–18] resp.). The PRISMA checklist is included in Additional file 1. Our review protocol is available upon request.

### Concept of collaborative goal setting

The concept of collaborative goal setting is still under development. For our review, we defined ‘collaborative’ as ‘an exchange of knowledge and information and/or cooperation between the professional(s) and the patient’ or as ‘a situation in which a patient is coached or supported by a professional’. Since the concept of collaborative goal setting within the framework of decision-making is still being developed, we also included studies that used similar terminology, like ‘mutual’ or ‘shared’. Moreover, as the distinction between ‘goal setting’ and ‘health priority setting’ is not always clear, both concepts were included in our search for relevant studies.

Since the concepts of collaborative goal setting and/or health priority setting in this context are under development, there are no established outcome measures. Therefore, we could not define all the relevant outcome measures up front. To avoid missing relevant studies, we included studies that reported on outcome measures reducible to collaborative goal setting and/or health priority setting. We did not report on the remaining outcomes of the included studies.

### Search strategy

We performed a systematic search in Pubmed, PsychInfo, CINAHL, Web of Science, Embase and the Cochrane Central Register of Controlled Trials, limited to publications in English and Dutch and including only the period from January 1990 to November 2015. The Pubmed search strategy, including search terms, is reported in Additional file 2. The study protocols obtained in the search were checked for published results. The reference lists in the reviews included in the search, as well as the reference lists of all included articles, were checked for possible missing studies.

### Study selection

Two researchers (NV and MH) screened titles and abstracts independently. The same researchers also selected the full texts independently. The following eligibility criteria were applied: randomised controlled trials (RCTs), non-randomised controlled trials (NRCTs), controlled before-after (CBA) studies, interrupted time series (ITS) and repeated measures studies. The population criterion was patients with multimorbidity or at least one chronic disease (mean age ± standard deviation incl. age 65). Both single and multifactorial interventions supporting collaborative goal setting or health priority setting were included. Included studies had to report on outcome measures reducible to collaborative goal setting and /or health priority setting.

### Data extraction and quality assessment

One investigator (MH) extracted study characteristics and outcomes from the included studies. These were checked by another investigator (NV). The data extraction form was based on EPOC’s ‘Data collection form: Intervention review – RCT and non-RCTs’ [16]. Risk of bias was assessed by two researchers (NV and MF) independently and then compared to evaluate the quality of the individual articles, according to the criteria for EPOC reviews [19].

### Data synthesis and analysis

Conducting a meta-analysis was not feasible because of the multifactorial character and variability of interventions. The results of the included studies were narratively analysed and interpreted.

## Results

**Fig. 1:**
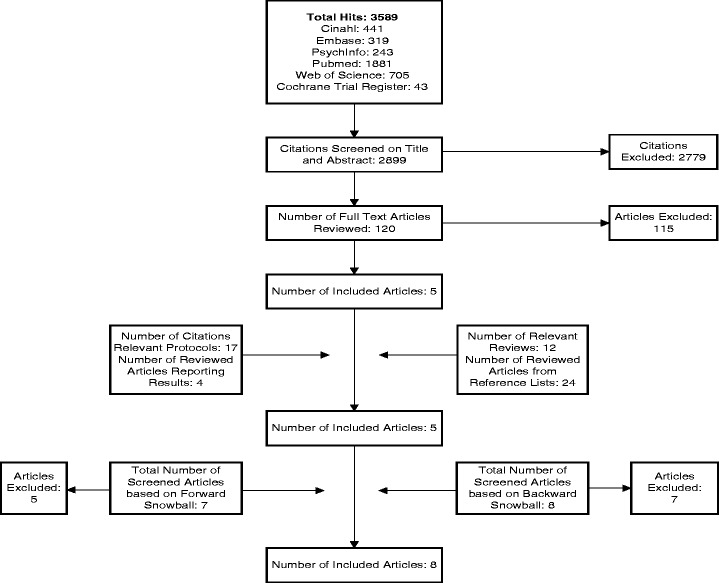
Flow Diagram of the Selection Procedure. This flow diagram is based on the PRISMA Flow Diagram 2009. It provides a summary of the different selection steps and indicates source, type of selection and numbers of inclusions and exclusions

A flow chart of the selection procedure is included in Fig. 1. The initial search identified 3589 citations. Based on the full-text assessment of 120 articles, five articles were included. The full-text assessment of the related articles about 17 study protocols did not result in any extra inclusions. Checking the references of 12 relevant reviews in the initial database search with additional full-text assessment of 24 articles did not result in any additional inclusions. Three articles were included based on the backward and forward reference checking of all included articles. The reasons to exclude full-text articles in relation to our eligibility criteria were study design (36), intervention (27), provider (13), multimorbidity or chronic condition (2), age (7), outcome (49) or a combination of criteria (45). Eventually, eight articles were included in this review.

### Risk of bias

**Fig. 2:**
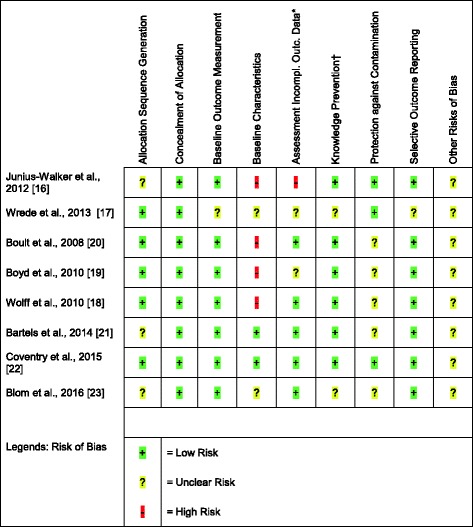
Risk of Bias of Included Studies. *Assessment of Incomplete Outcome Data. †Knowledge Prevention of Allocated Interventions. This figure summarises the risk of bias assessment of the articles included in this review. Risk assessment was based on criteria for EPOC reviews [16]. Allocation was adequately concealed (low risk) if the unit of allocation was by institution, team or professional, and allocation was performed on all units at the start of the study or if the unit of allocation was by patient or episode of care and a centralised randomisation scheme was used. The allocation sequence is adequately generated (low risk) if a random component in the sequence generation process is described. If there is no evidence of selective outcome reporting, this criterion is assessed as low risk. Baseline outcome measurements should show no important differences across study groups prior to the intervention (low risk). Baseline characteristics are assessed as low risk if reported and similar. Missing outcome measures should be unlikely to bias the results (low risk). Knowledge of the allocated intervention by assessors of primary outcome variables should be adequately prevented or the outcomes should be objective (low risk). The study was adequately protected against contamination if allocation was by community, institution or practice, and it is unlikely that the control group received the intervention. The ninth criterion is ‘other risks of bias’

All articles showed methodological concerns in varying degrees. The only risk of bias criterion that all studies scored ‘low risk’ on was allocation concealment. Four articles reported differences in the baseline characteristics of the intervention and control population, and two articles scored ‘unclear risk’ on this criterion. Five articles scored ‘unclear risk’ on protection against contamination. All the articles included ‘other risks of bias’ in the evaluation in the discussion; these risks (included in Additional file 3) were assessed as ‘unclear’ since their effects are unknown. All risks are summarised in Fig. 2. The elaborate risk assessment results that substantiate Fig. 2 are available upon request.

### Interventions in support of collaborative goal setting or health priority setting

The eight articles included in this review reported on five unique interventions. Based on these interventions, a distinction could be made between two articles reporting on the same underlying study on a single intervention concerning health priority setting, the PrefCheck [20, 21], and four multifactorial interventions in which goal setting and/or priority setting are part of a broader intervention. Three of the four multifactorial interventions described the same underlying study on the effects of Guided Care (GC) [22–24]. All the remaining articles dealt with distinct interventions, namely Helping Older People Experience Success (HOPES), the Collaborative Care Model and Integrated Systematic Care for Older People (ISCOPE) ([25–27] resp.).

Details of the interventions are summarised in Table 1. The PrefCheck (i.e. preferences in treatment planning for older patients) is the only included study that specifically focuses on shared priority setting between general practitioner (GP) and patient. In the PrefCheck intervention, a trained GP holds a consultation based on a specially developed guide, the PrefCheck. After the patient rates the importance of each existing health problem, the patient and GP exchange and document health and treatment priorities [20, 21].

**Table 1:** Interventions in individual studies

Study	Intervention
Junius-Walker et al., 2012 [20]	**Intervention-tool:** PrefCheck. The guide consists of the following: (1) Disclosure of the patient’s rating of the importance of each health problem in tabular form (2) A three-step guide to the exchange of health and treatment priorities in a patient-centred manner (3) A sheet on which to document priority health problems **Intervention:** 30-min GP training session in preparation for the priority setting consultations (use of an individual patient-related PrefCheck guide) by a research worker. A computer-aided geriatric assessment by a nurse after patient recruitment. Independent problem importance rating by GPs and patients, followed by a consultation using the PrefCheck. After 14 days, the second round of independent problem importance ratings, followed by a consultation using the PrefCheck. **Nature of desired change:** To improve shared health priority setting between GP and patient. **Timing:** One 30-min training session. Proximity to clinical decision-making is unclear. PrefCheck was given to the GP immediately before the consultation. 14-day follow-up period. **Comparison:** Standard practice control group (consultation using the patient’s problem list without importance ratings and PrefCheck)
Wrede et al., 2013 [21]	**Intervention:** Same as Junius-Walker et al., 2012 **Nature of desired change:** To improve shared health priority setting between GP and patient. **Timing:** 30-min GP training session in preparation for the priority setting consultations (use of an individual patient-related PrefCheck guide) by a research worker. A computer-aided geriatric assessment by a nurse, after patient recruitment. Independent problem importance rating by GPs and patients, followed by a consultation using the PrefCheck. Research on the first consultation. Follow-up consultations were not evaluated. **Comparison:** Standard practice control group (consultation using the patient’s problem list without importance ratings)
Boult et al., 2008 [24]	**Intervention:** Guided Care [37] A registered nurse completes an education programme and then uses a customised electronic health record (EHR) in working with 2 to 5 primary care physicians (PCP) to meet the complex needs of 50–60 older patients with multimorbidity. This Guided Care Nurse (GCN) has eight clinical activities: 1) Assessment: An initial assessment of the patient’s medical, functional, cognitive, affective, psychosocial, nutritional and environmental status during a visit at the patient’s home. The patient is asked to identify his or her highest priorities for optimising health and quality of life. 2) Planning: The EHR merges assessment data with evidence-based best practice recommendations to create a preliminary Care Guide. This preliminary Care Guide is adapted to reflect this individual patient by: 1) the GCN and the PCP, and 2) the GCN and the patient and caregiver. The final Care Guide summarises the patient’s status and plans to all professionals involved and is regularly updated by the GCN. A patient-friendly version (i.e. a lay version), called My Action Plan, is available in the patient’s home. 3) Chronic Disease Self-Management (CDSM): The patient’s self-efficacy in managing chronic conditions is promoted by referring him or her to a six-session CDSM course. 4) Monitoring: Monthly monitoring by telephone with reminders from the EHR to detect and address emerging problems. These problems are discussed with the PCP and appropriate action is taken. 5) Coaching: In conjunction with the monthly calls, the GCN uses motivational interviewing to reinforce the patient’s adherence to the Action Plan. 6) The GCN coordinates transitions between sites and care providers. 7) Educating and supporting caregivers. The GCN offers individual and group assistance to caregivers, consisting of initial assessment, a self-management course for caregivers, monthly support group meetings and ad hoc telephone consultations. 8) The GCN facilitates access to community resources. **Nature of desired change:** Initiation of the Guided Care Model to improve several aspects of health care quality for elderly patients with multimorbidity. **Timing:** Intervention duration 18 months, this article reports on results after 6 and 12 months. **Comparison:** Standard practice control group (usual care instead of guided care)
Boyd et al., 2010 [23]	**Intervention:** Guided Care **Nature of desired change:** Initiation of the Guided Care Model to improve several aspects of health care quality for elderly patients with multimorbidity. **Timing:** Intervention reports on 18 months. **Comparison:** Standard practice control group (usual care instead of guided care)
Wolff et al., 2010 [22]	**Intervention:** Guided Care Program for Family and Friends (GCPFF) The GCN: a) Makes an initial one-to-one assessment of the patient’s primary caregiver. b) Educates the caregiver and refers him or her to community resources. c) Offers ongoing ‘coaching’ to the caregiver. d) Facilitates six 90-min caregiver workshops based on the chronic disease self-management philosophy and approach. e) Facilitates one-hour-long unstructured monthly support group meetings. **Nature of desired change:** Initiation of the Guided Care Model to improve several aspects of health care quality for elderly patients with multimorbidity from the patient’s and caregiver’s perspectives. Improvement of caregiver depression, strain and productivity and their perceptions of the quality of patient care. **Timing:** Intervention reports on 18 months. **Comparison:** Standard practice control group (usual care instead of guided care)
Bartels et al., 2014 [25]	**Intervention:** Helping Older People Experience Success (HOPES) Combination of community living skills, social skills and healthy-living skills training with integrated preventive care coordinated by a nurse. The intervention contains a psychosocial element, facilitated by rehabilitation specialists; it consists of weekly skills training in group sessions over 1 year, followed by monthly booster sessions. In addition, two monthly community trips were organized to practise skills. The preventive element, HOPES Health Management, was facilitated by a nurse and consisted of monthly meetings to evaluate health care needs. Collaborative goal-setting is part of the health management component. Another step in the health management component is the completion of advance directives. **Nature of desired change:** Improvement of independent functioning and community tenure. **Timing:** 3 years: 1 year intensive phase, 1 year maintenance phase and 1 year follow-up. **Comparison:** Routine mental health services consisted of pharmacotherapy, case management or outreach by non-nurse clinicians, individual therapy, and access to rehabilitation services, such as groups and psychoeducation. Both intervention and control groups received these services.
Coventry et al., 2015 [26]	**Intervention:** Collaborative Care Model Eight psychological therapy sessions delivered by case managers who are ‘psychological well-being practitioners’. In the first treatment session, the psychological well-being practitioner uses a structured patient-centred interview to gather information and then introduces the patient to the standardised treatment manual and workbook to help develop a main problem statement and personalised goals. Two 10-min collaborative meetings (by telephone or in person) between the patient and the psychological well-being practitioner and a nurse from the patient’s general practice. Psychological well-being practitioners also work collaboratively with the patient and the practice nurse to monitor medication use. Use of established stepped care protocols. Psychological well-being practitioners received 5 days of training about the COINCIDE collaborative care model. Practice nurses followed a half-day workshop. Psychological well-being practitioners attended a weekly supervision session. **Nature of desired change:** Improvement of care access and quality. **Timing:** Eight brief face-to-face psychological therapy sessions (i.e. 30–45 min) within 3 months. Two collaborative meetings after sessions 2 and 8. Reports on results measured after 4 months. **Comparison:** Care as usual delivered by the general practitioner.
Blom et al., 2016 [27]	**Intervention:** The Integrated Systematic Care for Older PEople The GPs and nurses carrying out the intervention practices were trained in the delivery of proactive integrated care (e.g. in designing, conducting and adjusting a care plan). The GP or the practice nurse (under the supervision of the GP) created an integrated care, action and evaluation plan for participants with complex problems. Other care professionals were involved where needed (multidisciplinary consultation). The participant’s wishes and expectations about goals to be achieved were explored together with the informal caregiver(s). These priorities and goals were used as a starting point for making a care plan. **Nature of desired change:** The development of a care plan focusing on functioning for people with complex problems (i.e. a combination of somatic, functional, mental and social health problems). **Timing:** Two 3-h GP/practice nurse training sessions. Care plans for the first 10 patients per participating GP were made over a two- to three-month period. Follow-up period of 1 year. **Comparison:** Usual care. Participants receiving usual care were not included in the final analysis.

*GP* General Practitioner, *EHR* Electronic Health Record, *PCP* Primary Care Practice*, GCN* Guided Care Nurse, *CDSM* Chronic Disease Self-Management, *COINCIDE* The Collaborative Interventions for Circulation and Depression

Underlined: Goal-setting or priority-setting element

Although not designed as an intervention with a single focus on goal setting or health priority setting, the included multifactorial interventions show important similarities. In all these multifactorial interventions, there is an explicit focus on goal setting or priority setting, and goals or priorities are specifically determined. An important similarity is that they all were delivered primarily by a nurse or allied health professional (except for the ISCOPE intervention, which was provided by a GP or a nurse under GP supervision). Secondly, caregiver involvement was a common component in GC [22–24] and the ISCOPE intervention [27]. One of the GC studies focused on caregivers in particular [22]. Furthermore, training the professional providing the intervention was explicitly mentioned in GC [22–24], ISCOPE [27] and the Collaborative Care Model [26]. In addition, an educational programme for the patient involved was a common component in GC [22–24], the HOPES intervention [25] and the Collaborative Care Model [26]. Finally, explicit care planning was a common element in all the multifactorial interventions. Although there were common components as analysed above, these multifactorial interventions also showed considerable differences due to variability in the underlying model and study focus.

### Study and participant characteristics

One study used a randomised controlled trial design [25] and the remaining four were cluster randomised trials. In all the included studies, the intervention group was compared to the usual care or to standard practice. The number of study participants ranged from 42 to 1921 patients. One study focused on patients with a chronic disease, namely a serious mental illness [25]. One study recruited patients with diabetes and/or coronary heart disease who had also suffered from depressive symptoms for at least 2 weeks [26]. The remaining studies used a geriatric assessment [20, 21, 27] or Hierarchical Condition Category (HCC) [22–24] scores to determine multimorbidity. Although all the included studies satisfied our age criterion, two of them originally did not apply the ‘age 65 or older’ inclusion criterion for the underlying trial, but focused on broader age categories [25, 26]. Most of the studies were conducted in a general practice/primary care practice; one was conducted in a community mental health centre [25]. The studies were carried out in the USA [22–25], UK [26], Germany [20, 21] and the Netherlands [27]. The details of study and participant characteristics are summarised in Table 2.

**Table 2:** Study and participants’ characteristics

Study	Study design	Intervention	Participants		Outcomes
Junius-Walker et al., 2012 [20]	Cluster RCT	PrefCheck	Country	Germany	Doctor-patient agreement about the importance ratings of individual health problems Determination of priorities Rates of priority problem treatment planning
			Setting	GP/PCP	
			Number	322 participants	
			Condition	Multimorbidity^a^	
			Study age criterion	≥ 70 years of age	
			Study age	Intervention group: M = 76.9, no range reported Control group: M = 77.5, no range reported	
Wrede et al., 2013 [21]	Cluster RCT	PrefCheck	Country	Germany	Importance ratings discussed & prioritization at consultation level, health problems level and nature of the health problem level
			Setting	GP/PCP	
			Number	42 consultations	
			Condition	Multimorbidity^a^	
			Study age criterion	≥ 70 years of age	
			Study age	Intervention group: M = 77.0, IQR = 71.3–81.8 Control group: M = 78.0, IQR = 75.0–81.0	
Boult et al., 2008 [24]	Cluster RCT	Guided Care	Country	USA	PACIC^c^ goal-setting subscale
			Setting	GP/PCP	
			Number	904 participants	
			Condition	Multimorbidity^b^	
			Study age criterion	≥ 65 years of age	
			Study age	Intervention group M = 77.2, range 66–106 Control group M = 78.1, range 66–96	
Boyd et al., 2010 [23]	Cluster RCT	Guided Care	Country	USA	PACIC^c^ goal-setting subscale
			Setting	GP/PCP	
			Number	904 participants	
			Condition	Multimorbidity^b^	
			Study age criterion	≥ 65 years of age	
			Study age	Intervention group M = 77.2, range 66–106 Control group M = 78.1, range 66–96	
Wolff et al., 2010 [22]	Cluster RCT	Guided Care Program for Family and Friends (GCPFF)	Country	USA	PACIC^c^ goal-setting subscale ratings by caregivers
			Setting	GP/PCP	
			Number	308 caregiver-patient pairs	
			Condition	Multimorbidity^b^	
			Study age criterion	≥ 65 years of age (patient)	
			Study age	Intervention group (patient): M = 78.0, SD = 0.6 Control group: M = 77.9, SD = 0.7	
Bartels et al., 2014 [25]	RCT	Helping Older People Experience Success (HOPES)	Country	USA	Rate of completed advance directives
			Setting	Community mental-health agency	
			Number	183 participants	
			Condition	SMI	
			Study age criterion	≥ 50 years of age	
			Study age	Intervention group: M = 60.3, SD = 8.0 Control group: M = 60.1, SD = 7.1	
Coventry et al., 2015 [26]	Cluster RCT	Collaborative Care Model	Country	United Kingdom	PACIC^c^ goal-setting subscale
			Setting	GP/PCP	
			Number	387 participants	
			Condition	DM or CHD and depression^d^	
			Study age criterion	≥ 18 years of age	
			Study age	Intervention group: M = 57.9, SD = 12.0 Control group: M = 59.2, SD = 11.4	
Blom et al., 2016 [27]	Cluster RCT	The Integrated Systematic Care for Older PEople (ISCOPE) study	Country	The Netherlands	Median number and percentage of goals in care plans
			Setting	GP/PCP	
			Number	1921 participants	
			Condition	Multimorbidity*	
			Study age criterion	≥ 75 years of ages	
			Study age	Intervention group - not selected for care plan: M = 82.7, median = 79.2, IQR = 87.1 Intervention group-selected for care plan: M = 82.0, median = 78.8, IQR = 86.9 Control group: M = 83.7, median = 79.8, IQR = 88.0	

*ClusterRCT* Cluster Randomised Controlled Trial, *GP* General Practice, *PCP* Primary Care Practice*, M* mean, *IQR* interquartile range, *USA* United States of America, *PACIC* Patient Assessment of Chronic Illness Care, *SMI* serious mental illness, *SD* standard deviation*, DM* Diabetes Mellitus*, CHD* Chronic Heart Disease

^a^multimorbidity based on geriatric assessment

^b^multimorbidity based on Hierarchical Condition Category (HCC) scores

^c^Patient Assessment of Chronic Illness Care (PACIC) [28]

^d^Patients with diabetes mellitus and/or coronary heart disease who also had depressive symptoms for at least 2 weeks

### Effects on outcome measures

Two articles on the same trial applying the PrefCheck intervention reported on the rates of determined priorities [20, 21]. One article [20] only reported the determination and planning of priorities for the intervention group. Intervention GPs determined priorities together with 70% of patients. Treatment was planned for 84% of the priority problems and 37% of the non-priority problems. The PrefCheck intervention did not lead to an improvement in doctor-patient agreement about the importance of health problems [20].

The second article [21], based on 43 recorded consultations between 28 general practitioners and their patients, examined the effects of the PrefCheck intervention to determine the extent to which shared health priorities were set and facilitated through patient-centred behaviour. Twenty four consultations were held in the intervention group; the remaining 19 consultations belonged to the control group. General statements about setting priorities were made to clarify the purpose of prioritisation in 27.9% of all consultations (i.e. 12/43). It is not clearly stated whether these are intervention or control consultations. Six consultations held with the control group (*N* = 19) and nine consultations held with the intervention group (*N* = 24) addressed the importance of at least one health problem. No statistical significance of this outcome was reported [21]. At the health problems level (*N* = 216 health problems), an agreement on priority treatment was reached in only seven consultations (i.e. 3.2%). No agreements were made about setting priorities for everyday problems (*N* = 65) [21].

The rate of completed advance directives was considered an outcome measure for determined priorities and goals. The HOPES intervention increased the rate of completed advance directives in the intervention group versus the control group (61% versus 33%, effect size .59) [25].

The number of goals as part of a care plan was also considered a relevant outcome for our review. In the ISCOPE study, 288 participants were randomly selected to receive a care plan in which problems, goals and actions could be integrated. For 15% (*N* = 43) of them, a care plan was not prepared by the GP. In the interventional care plans, the median numbers of problems, goals and actions were the following: 3 (interquartile range (IQR) 2–4), 4 (IQR 2–5) and 3 (IQR 2–5), respectively [27]. We contacted the author to verify whether the numbers for the control group were also available. The author informed us that four patients who were not part of the selected group of 288 participants also received a care plan.

The Patient Assessment of Chronic Illness Care (PACIC) scale collects patient reports on the actions taken and the care received in line with the Chronic Care Model and intends to assess the patient-centred care received, with a focus on collaborative goal setting, problem solving and follow-up as key elements of self-management support [28]. In addition to an aggregate quality measurement, the PACIC scale consists of five subscales, i.e. goal setting, care coordination, decision support, problem solving and patient activation [24]. The PACIC’s ‘goal setting’ subscale is a relevant outcome measure for our review.

In the evaluation of the effects of the GC model, goal setting was considered to be ‘high quality’ when it occurred ‘most of the time’ or ‘always’ [24]. The studies applying the GC model and the Collaborative Care Model reported on the PACIC scale. In the GC model, the percentage of patients rating goal setting as ‘high quality’ after receiving care for 6 months was significantly higher for GC patients than for patients who received the usual care (i.e. 24.6% versus 11.6%, adjusted Odds Ratio (OR) 2.37, *p* < .001) [24]. Although no longer significant at the *p* < .05 level, the percentage of patients rating goal setting as ‘high quality’ after receiving care for 18 months was still higher for GC patients than for patients who received the usual care (i.e. 23.1% versus 15.3%, adjusted OR 1.53 (*p* = .005) [23].

In the Collaborative Care Model, patients’ scores on the goal setting subscale were higher in the collaborative arm than in the ‘usual care arm’ (mean 2.18 (SD 1.2) versus mean 1.77 (SD 1.0)) with an effect size of 0.37. This indicates that this care model was moderately effective in stimulating goal setting as an element of chronic care [26].

One article focused on the Guided Care Program for Family and Friends (GCPFF) [22] included caregiver reports that assessed the aggregate quality of chronic illness care provided to their care recipients by means of a modified version of the PACIC scale. On the goal setting subscale, quality ratings by caregivers in the GCPFF were significantly higher (mean 3.1 (Standard Error (SE) 0.13) versus mean 2.7 (SE 0.13)), with an effect size (ES) of 0.47 (95% confidence interval (ES) 0.15 to 0.79)).

## Discussion

Health care for elderly patients with a chronic health condition or multimorbidity may benefit from a switch from a disease-specific approach to a patient-goal-orientation [1–4]. Collaborative goal setting and/or health priority setting are necessary elements in this approach. This systematic review evaluates the effects of interventions supporting collaborative goal setting or health priority setting compared to usual care.

The possible benefits of a patient-goal orientation in care for elderly patients with a chronic disease or multimorbidity are increasingly recognised. However, compelling evidence for its benefits is lacking. Our review process and results made it retroactively clear that collaborative goal setting or health priority setting constitutes a relevant but ‘premature’ review topic. The review does, however, make a significant contribution to the further development of patient-goal-oriented health care in three areas: the concept of collaborative goal setting, single versus multifactorial interventions, and outcome measures and effects of collaborative goal setting or priority setting.

### The concept of collaborative goal setting

The concepts of ‘collaborative goal setting’ and ‘priority setting’ in this context are still under development. Moreover, our review illustrates that the distinction between them is not clear. In the evaluation of health priority setting in GC, the PACIC scale is used (i.e. a subscale on patients’ evaluation of goal setting). Priority setting can be considered part of goal setting or a separate, though related, concept. Within the framework of theory development as well as in the daily practice of care for elderly patients with multimorbidity, it is important to clearly define ‘priority setting’ and ‘goal setting’ and their mutual relation in the future.

An earlier systematic review addressed the evidence of complex interventions related to patient-goal-oriented health care, focusing on personalised care planning [29]. Our review differs from that review in two ways that are related to the concepts of ‘collaborative goal setting’ and ‘priority setting’. In Coulter’s review, goal setting is an element of personalised care planning, which includes action planning. Attainment of personal goals is a secondary outcome in this review. Only four of the 19 included articles reported on goal achievement. In the research implications, it is advised that future researchers examine the effects of personalised care planning on goal attainment, especially a patient’s personal goals as opposed to goals determined by clinicians or researchers. However, the concept and potential benefit of *collaborative* goals for clinical practice are not explicitly discussed in this review. Instead we focus on interventions concerning goal setting or priority setting as a *collaborative* process and aim to evaluate the effects of these collaborative goals and priorities. In addition, our review focuses on interventions supporting goal setting or priority setting without the limitation of a specific concept of care in the search strategy.

Within the framework of theory development as well as in the daily practice of care, it is also important to clearly define ‘shared decision-making’ and ‘goal setting’ and their mutual relation. For the time being, this mutual relation is not yet clearly defined. Goal setting is not an explicit element of the integrative definition of shared decision-making put forth by Makoul et al. [30]. Rose et al. [31] focused on shared decision-making *within* goal setting in rehabilitation settings. However, a ‘goal talk’ could also be viewed as a component of a shared decision-making process [32].

### Single versus multifactorial interventions

It follows from our study that single interventions regarding collaborative goal setting and/or priority setting are rare. They are usually components within varying multifactorial interventions. A systematic review of the related topic of the effectiveness on health outcomes of instrumental tools to assess patient treatment priorities and preferences within the framework of multimorbidity concluded that there is a lack of such tools, which is in line with our findings [33].

Considering the effectiveness of multifaceted or multifactorial interventions versus single-component interventions in changing health care professionals’ behaviour, an overview of systematic reviews showed that there is no compelling evidence that multifaceted interventions are more effective than single-component interventions [34]. However, the total effect of a multifaceted strategy depends on the effectiveness of its components and the interaction between them [35]. Based on the single-component character of a single intervention study only, it is too early to conclude that single interventions on health priority setting or collaborative goal setting would generally be ineffective. Nevertheless, in daily practice it is difficult to separate goal setting or priority setting from other care elements. Multifactorial interventions with an optimal mix of components seem to be the most promising in this phase of developing interventions that support goal setting or priority setting.

Our analysis of the multifactorial interventions found several common elements. Explicit care for goal setting or priority setting by a specific professional was one. However, there was variation in which person within the health care team provided the intervention. This could be a GP, a nurse, a GP and/or a practice nurse or a psychological wellbeing practitioner. This person could be part of the regular health care team or be introduced to the team based on the intervention. In addition, involvement of caregivers, training of intervention professionals, patient education and care planning were common elements in several or all interventions. However, these elements also showed considerable variability. Training of intervention professionals varied in time. The intervention duration and follow-up were also variable. Health priority setting and /or goal setting could be done in a separate consultation, could be the starting point of a broader care programme or be part of a preventive health management component. From this variability in content and use of generally common elements, it becomes clear that it is too early to give general recommendations for clinical practice at this stage, especially since we only found interventions in non-hospital settings. It could be useful to consider these aspects in further research and in the development of interventions including collaborative goal setting and/or priority setting.

### Outcome measures and effects of collaborative goal setting or priority setting

Despite the developmental phase of these interventions, we identified eight articles (i.e. seven cluster randomised and one randomised control trials) that described five unique interventions and relevant outcome measures that are reducible to collaborative goal setting and/or priority setting. The four multifactorial interventions had significant effects on the application of goal setting [22–24, 26], the number of advance directives [25] or led to the inclusion of problems, goals and actions in care plans [27]. The single intervention [20, 21] did not have a significant effect on doctor-patient agreement.

### Limitations

Identifying relevant articles in this broad topic area was challenging. Concepts and terminology are not always evident, and interventions are still under development. Most articles on integrated interventions do not focus on collaborative goal setting or priority setting, which may have led to our missing articles. We tried to prevent this by using a broad search terminology and a lengthy time period and by seeking additional information on articles and applying an extensive snowball procedure. Due to the restriction to publications in English and Dutch, potentially eligible articles in other languages may also have been excluded.

Due to a lack of established outcome measures, the relevant outcome measures could not be defined up front. To avoid missing relevant studies, we included studies that reported on outcome measures that are directly reducible to collaborative goal setting and/or health priority setting.

All the articles showed risks of bias in various degrees. This may be due to the behavioural character of the interventions and outcomes. The same limitation was described in systematic reviews on interventions in personalised care planning and patient-centred care, which conceptually overlap with patient-goal-oriented health care [29, 36].

Six of our reviewed articles dealt with four unique multifactorial interventions. Collaborative goal setting or priority setting constituted only one element of these interventions and their outcomes. It is impossible to draw clear conclusions on the effects of collaborative goal setting or priority setting within such a complex model, as other parts of the intervention may establish possible confounding effects. The included studies also report on different populations (as shown in Table 2), leading to difficulties when generalising results.

## Conclusions

To improve health care for elderly patients with chronic (multi)morbidity, it is inevitable to switch from a disease-specific approach to a focus on patient goals, including collaborative goal setting. A specific focus on collaborative goal setting and/or priority setting was mostly found in a multifactorial intervention, which seems to improve the application of goal setting and the numbers of agreed upon goals and advance directives. Although explicit care for goal setting or priority setting by a specific professional was a common element in the reviewed multifactorial interventions, it remains unclear which mix of key components makes the difference. Further research should determine the mix of essential elements within a multifactorial intervention to provide recommendations for daily practice. Conceptual clarity on collaborative goal setting and priority setting is a prerequisite for this. In addition, the evaluation of complex goal setting intervention studies is challenging and demands methodological innovation.

## Additional files

Additional file 1: PRISMA 2009 checklist includes the PRISMA 2009 checklist applying to this systematic review. (DOC 66 kb)

Additional file 2: Pubmed search strategy provides the Pubmed search strategy. (DOCX 17 kb)

Additional file 3: Other risks of bias provides an overview of ‘other risks of bias’ reported by the included studies’ authors in the discussion sections. (DOCX 20 kb)

## Abbreviations

CBA: Controlled before-after (studies)
EPOC: Effective Practice and Organization of Care
ES: Effect size
GC: Guided Care
GCPFF: Guided Care Program for Family and Friends
GP: General practitioner
HCC: Hierarchical condition category
HOPES: Helping Older People Experience Success
IQR: Interquartile range
ISCOPE: Integrated Systematic Care for Older PEople
ITS: Interrupted time series
MOOSE: Meta-analysis Of Observational Studies in Epidemiology
NRCT: Non-randomised controlled trials
OR: Odds ratio
PACIC: Patient Assessment of Chronic Illness Care
PRISMA: Preferred Reporting Items for Systematic Reviews and Meta-Analyses
RCT: Randomised controlled trial
SD: Standard deviation
SE: Standard error
UK: United Kingdom
USA: United States of America
