# Supplementary material for: Collaborative goal setting with elderly patients with chronic disease or multimorbidity: a systematic review
Source: BMC Geriatr. 2017 Jul 31;17:167. doi: 10.1186/s12877-017-0534-0 (PMC5537926; doi:10.1186/s12877-017-0534-0)
Supplement: Supplementary file 2 — Pubmed search strategy provides the Pubmed search strategy. (DOCX 17 kb) [file 12877_2017_534_MOESM2_ESM.docx]

**Additional File 2: Pubmed search strategy**

Date search: 02-12-2015

Hits: 1881

| 1 | aged[mesh] |
| --- | --- |
| 2 | older[tiab] |
| 3 | elderly[tiab] |
| 4 | geriatr*[tiab] |
| 5 | 1 OR 2 OR 3 OR 4 |
| 6 | "Chronic Disease"[Mesh] |
| 7 | chronic disease*[tiab] |
| 8 | 6 OR 7 |
| 9 | "Comorbidity"[Mesh] |
| 10 | multimorbid*[tiab] |
| 11 | multimorbid*[tiab] |
| 12 | multi morbid*[tiab] |
| 13 | comorbid*[tiab] |
| 14 | co-morbid*[tiab] |
| 15 | co morbid*[tiab] |
| 16 | 9 OR 10 OR 11 OR 12 OR 13 OR 14 OR 15 |
| 17 | 8 OR 16 |
| 18 | "Patient-Centered Care"[Mesh] |
| 19 | "Patient Care Planning"[Mesh] |
| 20 | 18 OR 19 |
| 21 | "Decision Making"[Mesh]) |
| 22 | 20 AND 21 |
| 23 | shared[tiab] |
| 24 | decision making[tiab] |
| 25 | decision-making[tiab] |
| 26 | decisionmaking[tiab] |
| 27 | goal[tiab] |
| 28 | goals[tiab] |
| 29 | 24 OR 25 OR 26 OR 27 OR 28 |
| 30 | 23 AND 29 |
| 31 | 22 OR 30 |
| 32 | goal setting[tiab] |
| 33 | mutual goal*[tiab] |
| 34 | patient engagement[tiab] |
| 35 | collaborative partner*[tiab] |
| 36 | informing[tiab] |
| 37 | listening[tiab] |
| 38 | reasoning[tiab] |
| 39 | negotiating[tiab] |
| 40 | patient centered[tiab] |
| 41 | patient-caregiver relationship[tiab] |
| 42 | patient care planning[tiab] |
| 43 | advance care planning[tiab] |
| 44 | care plan[tiab] |
| 45 | care plans[tiab] |
| 46 | care-plan[tiab] |
| 47 | care-plans[tiab] |
| 48 | goal attainment scaling[tiab] |
| 49 | goal oriented[tiab] |
| 50 | optimal care[tiab] |
| 51 | goal-oriented approach[tiab] |
| 52 | goal-orientation[tiab] |
| 53 | goal orientation[tiab] |
| 54 | health priorities[tiab] |
| 55 | holistic approach[tiab] |
| 56 | priority setting process[tiab] |
| 57 | self management[tiab] |
| 58 | self-management[tiab] |
| 59 | collaborative goal-setting[tiab] |
| 60 | collaborative goal setting[tiab] |
| 61 | collaborative goal[tiab] |
| 62 | collaborative goals[tiab] |
| 63 | 31 OR 32 OR 33 OR 34 OR 35 OR 36 OR 37 OR 38 OR 39 OR 40 OR 41 OR 42 OR 43 OR 44 OR 45 OR 46 OR 47 OR 48 OR 49 OR 50 OR 51 OR 52 OR 53 OR 54 OR 55 OR 56 OR 57 OR 58 OR 59 OR 60 OR 61 OR 62 |
| 64 | English [Language] |
| 65 | Dutch[Language] |
| 66 | 64 OR 65 |
| 67 | ("1990/01/01"[Date - Publication] : "3000"[Date - Publication]) |
| 68 | 17 AND 63 |
| 69 | 68 AND 66 |
| 70 | 69 AND 67 |
| 71 | 5 AND 70 |
