# Supplementary material for: Collaborative goal setting with elderly patients with chronic disease or multimorbidity: a systematic review
Source: BMC Geriatr. 2017 Jul 31;17:167. doi: 10.1186/s12877-017-0534-0 (PMC5537926; doi:10.1186/s12877-017-0534-0)
Supplement: Supplementary file 3 — Other risks of bias provides an overview of ‘other risks of bias’ reported by the included studies’ authors in the discussion sections. (DOCX 20 kb) [file 12877_2017_534_MOESM3_ESM.docx]

**Additional File 3:** **Other risks of bias**

| **Author** | **Other risks of bias as reported by the authors** |
| --- | --- |
|  |  |
| Junius-Walker et al., 2012 [20] | - Shortness of consultation time and doctors’ training - Re-evaluation of on average 12 problems per patient could be difficult for participating practitioners to achieve |
|  |  |
| Wrede et al., 2013 [21] | - Small numbers of participants and audio recordings - Lack of conceptual clarity around terms like prioritisation and patient-centredness - Shortness of consultation time and doctors’ training |
|  |  |
| Boult et al., 2008 [24] | - Small number of nurses (7) - Reliance on self-reported data - Limited consent rate - Geographic scope (urban mid-Atlantic states in the USA) |
|  |  |
| Boyd et al., 2010 [23] | - Only 38% of high-risk patients consented to participate - Less complete entry of diagnoses by practices - Proxies’ ratings of the PACIC were accepted - Assumption of a common treatment effect across teams within each practice |
|  |  |
| Wolff et al., 2010 [22] | - Multifaceted nature of the intervention - Heterogeneity in patient and caregiver characteristics - Uncertainty about the extent to which nurses implemented the model - Power of the study is based on its ability to detect changes in patient rather than caregiver outcomes - Relatively small number of caregivers - Inability to disentangle which components of the intervention did or did not work or were most salient to patients and caregivers - Unclear which outcomes are most salient to caregivers |
|  |  |
| Bartels et al., 2014 [25] | - Study sample was predominantly white - Attribution of study outcomes to intervention components - Needed more targeted disease management and longer follow-up period to demonstrate improved health outcomes |
|  |  |
| Coventry et al., 2015 [26] | - Short follow-up period - Self-reported data on the use of antidepressants - General practitioners in both arms were notified that participants met criteria for depression - No collection of objective measures of physical functioning |
|  |  |
| Blom et al., 2016 [27] | - Non-responders were slightly more vulnerable - No data on fidelity to the care plan - No repeated assessments over a longer period |

*GP* General Practitioner, *HCC* Hierarchical Condition Category, *PACIC* Patient Assessment of Chronic Illness Care
